# Harnessing the immunotherapeutic potential of CDK4/6 inhibitors in melanoma: is timing everything?

**DOI:** 10.1038/s41698-022-00273-9

**Published:** 2022-04-20

**Authors:** Emily J. Lelliott, Karen E. Sheppard, Grant A. McArthur

**Affiliations:** 1 Cancer Research Division, Peter MacCallum Cancer Centre, Melbourne, VIC Australia; 2 Sir Peter MacCallum Department of Oncology, University of Melbourne, Parkville, VIC Australia; 3 Department of Biochemistry and Pharmacology, University of Melbourne, Parkville, VIC Australia

**Keywords:** Cancer immunotherapy, Translational research

## Abstract

CDK4/6 inhibitors (CDK4/6i) were developed as a cancer therapeutic on the basis of their tumor-intrinsic cytostatic potential, but have since demonstrated profound activity as immunomodulatory agents. While currently approved to treat hormone receptor-positive breast cancer, these inhibitors are under investigation in clinical trials as treatments for a range of cancer types, including melanoma. Melanoma is a highly immunogenic cancer, and has always been situated at the forefront of cancer immunotherapy development. Recent revelations into the immunotherapeutic activity of CDK4/6i, therefore, have significant implications for the utility of these agents as melanoma therapies. In recent studies, we and others have proven the immunomodulatory effects of CDK4/6i to be multifaceted and complex. Among the most notable effects, CDK4/6 inhibition induces transcriptional reprogramming in both tumor cells and immune cells to enhance tumor cell immunogenicity, promote an immune-rich tumor microenvironment, and skew T cell differentiation into a stem-like phenotype that is more amenable to immune checkpoint inhibition. However, in some contexts, the specific immunomodulatory effects of CDK4/6i may impinge on anti-tumor immunity. For example, CDK4/6 inhibition restricts optimal T cells expansion, and when used in combination with BRAF/MEK-targeted therapies, depletes immune-potentiating myeloid subsets from the tumor microenvironment. We propose that such effects, both positive and negative, may be mitigated or exacerbated by altering the CDK4/6i dosing regimen. Here, we discuss what the most recent insights mean for clinical trial design, and propose clinical considerations and strategies that may exploit the full immunotherapeutic potential of CDK4/6 inhibitors.

Small molecule-inhibitors of cyclin-dependent kinases 4 and 6 (CDK4/6) were initially developed as a therapeutic to block proliferation of tumor cells, specifically targeting tumor types with two key features. The first is the aberrant activity of CDK4/6 itself; this can be due to various factors such as overexpression of the CDK4/6 binding partner, Cyclin D1, and/or deletion of the endogenous CDK4/6 inhibitor, p16^INK4a^^1,2^. The second is the functional integrity of the tumor suppressor retinoblastoma protein (RB); the most eminent CDK4/6 substrate, which is activated following CDK4/6 inhibition^3–5^. Melanoma is an attractive target for CDK4/6 inhibitors (CDK4/6i) as the p16^INK4a^/Cyclin D1-CDK4/6/RB pathway is dysregulated in a majority of melanomas, while mutations causing loss or dysfunction of RB itself are uncommon^6,7^. This has prompted clinical trials incorporating CDK4/6i into existing therapeutic protocols for melanoma, for example by combining CDK4/6i with current standard-of-care targeted inhibitors of oncogenic BRAF and/or MEK (NCT01719380, NCT01777776, NCT04720768)^8^.

In addition to the tumor-intrinsic therapeutic potential of CDK4/6i, a surge of recent studies has uncovered profound and unexpected immunomodulatory effects of these cell cycle inhibitors^9–21^. Such discoveries have highlighted the immunotherapeutic potential of CDK4/6i, which has significant implications for the utility of these agents for treating melanoma. Indeed, the high mutational burden and resulting neoantigen profile of melanoma have placed it at the forefront of immunotherapeutic development, and immune engagement appears to be a significant factor underpinning the success of existing melanoma therapies^22^. This is not only the case for immune checkpoint inhibitors (ICIs), which directly derepress anti-tumor T cell immunity, but also for targeted BRAF and MEK inhibitors, the efficacy of which has been at least partly attributed to immune engagement^23–28^. We and others have recently shed new light on the immunomodulatory effects of CDK4/6i, both alone and in combination with current melanoma standard-of-care therapies, including ICI (targeting PD-1, PD-L1, and CTLA-4) and targeted BRAF and MEK therapies^9,12,15–19^. These studies provide new translational and mechanistic insights into the prospective use of CDK4/6i as immunomodulatory clinical tools. Here we discuss these most recent insights and propose CDK4/6i dosing strategies that may maximize the immunotherapeutic activity of these inhibitors.

## Boosting response rates to ICI

ICIs are used widely as a standard-of-care therapy for melanoma, with an encouraging five-year overall survival rate of around 50%^29^. Still, a large proportion of patients fail to achieve any long-term benefit from ICI, through mechanisms that remain incompletely understood. Interestingly, CDK4/6i significantly enhances the anti-tumor efficacy of PD-1/PD-L1 checkpoint inhibition in numerous preclinical mouse models^9–12,14,16^. The synergy of this combination has been largely attributed to tumor-intrinsic immunomodulatory activity of CDK4/6 inhibitors; for example through altering MHC I and PD-L1 expression and increasing tumor cell production of T cell chemoattractants^9,11,13,15^. This hypothesis was strengthened recently when a meta-analysis of over 1000 patients identified amplification of *CCND1* (encoding Cyclin D1) as one of the strongest tumor-intrinsic predictors of resistance to ICI^30^. Given a key function of Cyclin D1 is to mediate activation of CDK4/6, it is reasonable to postulate that shutting down this pathway with CDK4/6i would reverse the corresponding ICI resistance phenotype. Indeed, an earlier study by Jerby-Arnon and colleagues demonstrated evidence of this reversal, with CDK4/6i abrogating a tumor-intrinsic transcriptional program associated with T cell exclusion and ICI resistance in melanoma^14^. Importantly, the induction of this transcriptional signature was seen in melanoma cell lines in vitro. Hence, while it is possible that CDK4/6 overactivity may indirectly contribute to immune exclusion by driving aggressive tumor growth, the direct effects of CDK4/6 inhibition on transcriptional programs associated with ICI resistance highlights a stand-alone function of CDK4/6 in promoting immune evasion.

In addition to immunomodulation through tumor-intrinsic mechanisms, CDK4/6i also modulates anti-tumor immunity through direct effects on T lymphocytes. Specifically, CDK4/6 inhibition potently attenuates the proliferation of T regulatory cells (Tregs), relieving immunosuppression in the tumor microenvironment, and has also been shown to enhance activation of effector T cells through the depression of nuclear factor of activated T cells (NFAT) transcription factors^9,10,16,18^. Furthermore, somewhat akin to the discovery of CDK4/6i-mediated transcriptional reprogramming in tumors described by Jerby-Arnon and colleagues^14^, we and others recently demonstrated similarly profound transcriptional reprogramming in lymphocytes exposed to CDK4/6i^16,17^. Specifically, CDK4/6 inhibition directly altered differentiation of CD8+ cytotoxic T lymphocytes (CTLs), enhancing the stem or memory-like properties of these cells^16,17^. This was an important discovery as stem-like CTLs are implicated as the key intratumoral T cell subset underpinning clinical responses to ICI^31–33^. Indeed, in melanoma patients, the specific CTL-intrinsic gene signature induced by CDK4/6i correlates strongly with favorable responses to anti-PD-1/L1 and anti-CTLA-4 therapy^16^. These recent findings underscore an important new mechanism of ICI + CDK4/6i synergy, whereby CDK4/6 inhibition promotes a more favorable intratumoral T cell pool for immune checkpoint targeting.

The potent immunomodulatory effects of CDK4/6i have prompted clinical trials evaluating CDK4/6i in combination with ICI for the treatment of various cancer types (NCT02791334, NCT04118036, NCT02778685, NCT04075604, NCT02779751). However, it is likely that trial design will be paramount in realizing the true benefit of this combination. In a series of preclinical mouse experiments, Schaer and colleagues demonstrated the most efficacious schedule for this combination was continuous administration of a CDK4/6 inhibitor before, throughout, and after anti-PD-L1 therapy, based on the rationale that CDK4/6 inhibition promotes and maintains a T cell-inflamed microenvironment^12^. As a result of this study, a clinical trial was established to evaluate this combination and specific schedule^34^. Unfortunately, however, a 14 day lead-in period with the CDK4/6i, abemaciclib, prior to the addition of anti-PD-L1, was deemed intolerable, with dose-limiting immune-related hepatotoxicity occurring in 3 out of 4 patients with breast, small cell lung, and esophageal cancer (NCT02791334)^34^. Notably, the impact of CDK4/6i on immunotoxicity reinforces the immunomodulatory effects of CDK4/6 inhibition in patients. Interestingly, in this same trial, no dose-limiting or liver toxicities were observed with this combination in the absence of a CDK4/6i lead-in period^34^. While this offers some promise in regard to the toxicity profile of combination treatment, a similar trial examining upfront continuous administration of abemaciclib and the PD-1 inhibitor, pembrolizumab, reported serious toxicity in patients with hormone receptor-positive (HR+) breast and non-small cell lung cancer (NCT02779751)^35,36^. Encouragingly, however, grade 3/4 adverse events were reversible following drug holds and corticosteroid treatment^36^. These adverse events are unlikely due to the choice of ICI (i.e. a PD-1 versus a PD-L1 inhibitor), as a further phase I/II trial for HR + breast cancer, examining upfront combination treatment with the CDK4/6i, palbociclib (given on a 3 week on/1 week off schedule), with pembrolizumab and letrozole, was well tolerated and reportedly safe^37^. It is worth noting however, approximately 50% of patients in this trial required dose delays or dose reductions to manage toxicities associated with neutropenia^37^, which is a common side effect of CDK4/6i^38–40^. Indeed, the principal toxicity seen across all 3 approved CDK4/6 inhibitors (abemaciclib, ribociclib and palbociclib) is hematological, including neutropenia, anemia and thrombocytopenia^38–40^. This is unlikely to be avoided with strategic choice of CDK4/6 inhibitor, or development of new CDK4/6 inhibitors, as these toxicities are considered to be on-target effects, with reduced production of blood cells resulting from CDK4/6i-mediated inhibition of progenitor cell proliferation. Rather, the most effective strategy to minimize hematological toxicities with these agents will be to reduce the duration of CDK4/6i, thereby allowing sufficient periods for cell production to recover.

Despite overlap in on-target hematological toxicities, it is important to note that approved CDK4/6i have reportedly different pharmacological activity and overall toxicity profiles^41–43^, which may facilitate treatment choice, particularly in ICI combination regimens. For example, palbociclib and ribociclib have relatively lower off-target toxicities (notably gastrointestinal) than abemaciclib, while the frequency of hepatotoxicity is highest for ribociclib^44^. Given immune-related hepatotoxicity is also common with ICI^45^, selecting a CDK4/6i agent and dosing schedule that minimizes liver toxicities will be important. In light of the pharmacological heterogeneity of CDK4/6i, it is also essential to reflect on possible variations in the immunomodulatory activity of each agent when considering the most suitable CDK4/6i to combine with ICI. So far, key immune-related effects of CDK4/6i reported preclinically have been consistent across at least two agents. These include effects on tumor immunogenicity (abemaciclib, palbociclib)^9^, Treg depletion (abemaciclib, palbociclib, trilaciclib)^9,10,16,18^ and CTL differentiation (palbociclib, abemaciclib, ribociclib, trilaciclib)^10,16–18^. However, these may well be acute effects of CDK4/6 inhibition and it will be worthwhile monitoring for differences between agents over the medium to long term. Medium to long-term effects may be impacted by pharmacological affinities for other CDKs, which could otherwise mediate resistance to CDK4/6i, and which vary between inhibitors (perhaps most notable is inhibition of CDK1 and CDK2 by abemaciclib^41^).

Even in settings where toxicities are manageable, concurrent and continuous administration of CDK4/6i with ICI may prove to be a double-edged sword in regard to anti-tumor activity. Consistent with the tumor-intrinsic cytostatic effects of CDK4/6i, these inhibitors also dampen proliferation of CTLs via the activation of RB in these cells^16^. Expansion of tumor-specific CTLs is fundamental to the efficacy of ICI, and it is likely this expansion will be somewhat compromised while CDK4/6i is administered. However, the CDK4/6i-mediated acquisition of stem-like properties in CTLs is, at least in part, regulated via RB^16^, and hence slowing the cell cycle in itself may very well be critical for this transcriptional reprogramming to take place. As such, balancing the anti-proliferative effects of CDK4/6i on both tumors and lymphocytes in a way that induces tumor cell cytostasis, without compromising anti-tumor T cell immunity, will be important to maximize the clinical activity of CDK4/6i+ICI combinations.

Currently, CDK4/6i are most often administered either continuously (abemaciclib) or on a 3 week on/1 week off schedule (palbociclib/ribociclib). However, CDK4/6i-mediated transcriptional reprogramming occurs within lymphocytes and tumors in as little as 24 h and 1 week, respectively^14,16^. It may therefore be worthwhile investigating CDK4/6i+ICI combinations with shorter intermittent periods of CDK4/6i treatment. In addition to reducing the potential for CDK4/6i-driven hematological toxicities, a shorter CDK4/6i schedule would be sufficient to induce transcriptional changes that facilitate immune engagement, while allowing longer periods for CTLs to expand efficiently to carry out their anti-tumor effector function. It is promising to note that following cessation of CDK4/6i treatment, therapy-induced transcriptional reprogramming in T cells is stable for up to 30 days in mice^16^; although further studies are needed to determine the stability of CDK4/6i-induced transcriptional changes in tumor cells. At the level of the tumor immune microenvironment, just a single treatment with CDK4/6i is sufficient to decrease the frequency of both CTLs and Tregs within 24 hours in mice^21^. Notably however, CTLs rapidly recover within 48 hours, while Tregs remain depleted for up to one week^21^. Together these recent findings suggest that shorter intervals of CDK4/6i treatment would, at a minimum, be sufficient to improve the anti-tumor T cell phenotype, and maintain a favorable distribution of effector/regulatory T cell subsets within tumors.

In contrast to a shorter intermittent CDK4/6i dosing regimen throughout ICI treatment, another potential strategy would be preconditioning tumors with CDK4/6i, and ceasing this treatment prior to starting ICI. Such an approach would see the benefits of CDK4/6i-mediated transcriptional priming of T cells and tumor cells, while again potentially avoiding toxicities associated with continued CDK4/6i and ICI co-treatment. Further, using CDK4/6i solely as a priming tool removes the potential complication of restricted T cell expansion following ICI, which is likely to occur if CDK4/6i is continuously administered. In support of this approach, we recently showed that transient CDK4/6i-priming is sufficient to sensitize anti-PD-1-refratory tumors to subsequent anti-PD-1 therapy in mice, without the need for continual CDK4/6i treatment^16^. Excitingly, there is also evidence of this occurring in melanoma patients. Indeed, in one case study, we found that CDK4/6i treatment induced a T cell-intrinsic stem-like transcriptional signature, which preceded a clinical and immunological response to subsequent treatment with anti-CTLA-4 + anti-PD1 therapy^16^. A further case study also described a patient previously refractory to anti-PD-1 checkpoint blockade, who later achieved a sustained and near complete response to this therapy after interim treatment with a CDK4/6 inhibitor^46^. Together this suggests that CDK4/6i may be used to sensitize otherwise resistant patients to ICI, likely in part through favorable priming of the intratumoral T cell pool. However, whether transient short-term CDK4/6i is also sufficient to promote a prolonged tumor-intrinsic ICI-responsive transcriptional program requires further investigation.

## Augmenting the immunological effects of BRAF and MEK inhibitors

Like CDK4/6i, BRAF and MEK inhibitors (BRAFi + MEKi), which inhibit the MAPK/ERK pathway, were originally developed to block tumor cell proliferation and survival, but were subsequently discovered to augment anti-tumor immunity^23–28^. Most notably, BRAF + MEK inhibition is associated with an influx of T cells into the tumor microenvironment in patients^23,25,28^. Mechanistically, this is suggested to arise from therapy-induced tumor immunogenic cell death, which promotes the activation of dendritic cells (DCs) and subsequent recruitment of T cells and other inflammatory immune subsets to the tumor site^28^. Notably, this immune infiltrate is diminished in tumors that progress on BRAFi+MEKi^23,28^. Whether this immune exclusion is simply a bi-product of tumor progression driven by tumor-intrinsic resistance to BRAFi+MEKi, or is in fact itself a critical factor driving tumor progression is unclear. Most likely, both scenarios form a feedforward loop, cooperating simultaneously to accelerate tumor progression. Indeed, among the many genes upregulated downstream of MAPK/ERK signaling is *CCND1*^47,48^. It may therefore be the case that BRAFi+MEKi-mediated inhibition of *CCND1* transcription enhances tumor immunogenicity through mechanisms similar to that of CDK4/6 inhibition. Likewise, tumor-intrinsic BRAFi+MEKi resistance via reactivation of the MAPK/ERK pathway may lead to tumor immune exclusion via increased expression of *CCND1* and subsequent CDK4/6 activation, which is associated with a tumor-intrinsic T cell exclusionary transcriptional program^14^. As such, BRAFi+MEKi therapy resistance not only reinstates tumor cell proliferation and survival, but may simultaneously dampen immune-mediated tumor control through a complex network of tumor-intrinsic transcriptional changes, that could be partly driven by an upregulation of Cyclin D1.

The convergence of MAPK/ERK signaling with CDK4/6 activity makes co-targeting these pathways an attractive clinical approach for two reasons. Firstly, this combination (i.e. CDK4/6i plus BRAFi and/or MEKi) potently suppresses tumor proliferation by maintaining RB in an activated, hypophosphorylated state^49,50^. Secondly, this approach should, theoretically, maintain tumor immunogenicity though blocking immune evasive transcriptional programs associated with upregulation of Cyclin D1 in BRAFi+MEKi resistant cells. However, the optimal strategy to employ CDK4/6i as immunomodulatory agents in this combination remains in question. We and others have shown that combined inhibition of CDK4/6 and MAPK/ERK does indeed enhance anti-tumor T cell immunity in mice through select mechanisms that align with the known immunomodulatory effects of the individual therapeutic agents, such as inducing immunogenic tumor cell death, enhancing T cell memory, and reducing Treg frequencies in tumors^18,19^. However, in addition to these favorable effects, continuous co-treatment with BRAFi, MEKi and CDK4/6i potently depletes melanoma tumors of myeloid cells, including immune-potentiating subsets, such as chemokine-producing macrophages and cross-priming CD103+ DCs^18^. Importantly, these myeloid subsets are critical for ongoing recruitment of T cells to the tumor microenvironment, as well as simulating T cells that enter the tumor^51,52^. Alas, while combined BRAF + MEK + CDK4/6 inhibition initially triggers a strong T cell response, it also depletes the immune subsets required for maintaining this response over the medium to longer term. Indeed, mice and melanoma patients with low frequencies of these tumor-associated myeloid subsets respond less favorably to anti-PD-1 and anti-CTLA-4 ICI and have worse overall clinical outcomes^18,51^. Perhaps a promising observation is that depletion of these subsets in mice is not immediate, but rather occurs after several days to a week of continuous treatment^18^. In contrast, therapy-induced depletion of myeloid-derived suppressor cells (MDSCs) occurs rapidly, within as little as 2 days of treatment^18^. Notably, MDSCs have a very short lifespan, and their presence in tumors relies on constant recruitment to the tumor site^53^. The rapid depletion of these MDSCs, in comparison to longer-lived macrophages and DCs, suggests that therapy-induced depletion of myeloid subsets occurs through a hindrance in their production. Certainly, this is consistent with the known on-target hematological toxicities observed with CDK4/6i in the clinic. It is therefore possible that shorter intermittent scheduling of this therapy combination, in a way that limits the hematological impacts of CDK4/6i, may selectively deplete suppressive myeloid subsets, while protecting the immune-potentiating subsets that support T cell immunity. Of course, an intermittent CDK4/6i dosing schedule may also be an efficacious strategy to optimize anti-tumor T cell immunity for the additional aforementioned reasons. Given how rapidly CDK4/6 inhibition induces transcriptional programming, it is possible that even a once-off treatment of CDK4/6i given upfront with BRAFi+MEKi would be sufficient to enhance the quality of the anti-tumor T cell response initiated by BRAFi-MEKi, without disrupting immune-potentiating myeloid populations or compromising ongoing CTL expansion. However, from a tumor-intrinsic perspective, whether shortening the CDK4/6i dosing schedule would significantly compromise the tumor-intrinsic efficacy of BRAFi/MEKi+CDK4/6i, in regards to maintaining RB activation, requires further investigation. There is certainly some evidence in vitro that the anti-proliferative effects of BRAFi+CDK4/6i is maintained for up to a week following the cessation of CDK4/6i^49^, though this needs to be examined in vivo, and over a longer period of time. Interestingly, in immunodeficient mouse models, intermittent dosing with CDK4/6i in a MEKi+CDK4/6i combination actually outperforms the continuous schedule by delaying the onset of therapy resistance^54^. Indeed, there are other examples where intermittent scheduling of targeted therapies has demonstrated superior tumor-intrinsic activity compared to the continuous schedule^55,56^. It is therefore possible that intermittent CDK4/6i scheduling would potentially have beneficial impacts at both an immune and tumor-intrinsic level.

Combined inhibition of BRAF, MEK and CDK4/6 has shown considerable efficacy in preclinical studies^18,49^, and is now being evaluated in clinical trials using a schedule of continuous BRAFi+MEKi with concurrent CDK4/6i on a 3 week on/1 week off 28 day cycle (NCT01543698, NCT04720768, NCT02159066). Despite significant biological rationale supporting this combination, so far in early phase trials the triple combination has not improved response rates compared to dual BRAFi+MEKi (NCT01543698)^8^. Given the recently discovered and profound effects of CDK4/6i on anti-tumor immunity, as clinical trials progress in this area it is important to reflect on the potential caveats of a continuous CDK4/6i schedule.

## Closing remarks

CDK4/6 inhibitors have the potential to augment immune-mediated therapeutic responses to existing standard-of-care treatments for melanoma, including both ICI and BRAF/MEK-targeted therapies. However, clinical trial design in regard to dose timing and patient cohort selection will likely be essential in realizing the benefit of these novel combinations. Currently, biomarkers for selecting patients for CDK4/6i treatment are focussed on tumor-intrinsic factors, such as RB status. However, in light of the profound immunotherapeutic activity of CDK4/6i, additional immune biomarkers should be considered. For example, the presence of intratumoral MDSCs or Tregs, or a high neutrophil-to-lymphocyte ratio (associated with poor ICI responses^57,58^), may identify opportunities to alleviate immunosuppression using CDK4/6i.

It will also be critical to consider patient eligibility criteria for combination trials, and determine at what stage to assign patients to a CDK4/6i combination. Upfront CDK4/6i combinations, including alternative scheduling regimens, might prove both efficacious and biologically insightful in the neoadjuvant setting, where both ICI and BRAF/MEK-targeted therapies have already shown some success^59,60^. Given BRAFi+MEKi-mediated tumor cell death induces a T cell response, upfront co-treatment with CDK4/6i may be used to opportunistically improve the quality of this immune response early on. Importantly, short-term CDK4/6i in this setting may be sufficient, and even optimal, to augment and prolong the BRAFi+MEKi-mediated T cell response by promoting T cell memory differentiation following priming by DCs. Similarly, short-term CDK4/6i dosing either prior to or intermittently throughout ICI is a potential strategy to deplete Tregs, while optimizing long-term CTL responses. Importantly, patients who have previously failed ICI therapy should be considered as eligible for CDK4/6i-ICI combination trials, given the capacity for CDK4/6i to directly induce both tumor- and CTL-intrinsic transcriptional signatures associated with favorable ICI responses. Indeed, recent findings, both preclinically and in patients, suggest CDK4/6i may in fact sensitize otherwise resistant tumors to ICI. As such, patients who have previously failed ICI may stand to benefit the most from this combination.

## Data Availability

Data sharing not applicable to this article as no datasets were generated or analyzed
